# Increased efficiency of *Campylobacter jejuni N*-oligosaccharyltransferase PglB by structure-guided engineering

**DOI:** 10.1098/rsob.140227

**Published:** 2015-04-01

**Authors:** Julian Ihssen, Jürgen Haas, Michael Kowarik, Luzia Wiesli, Michael Wacker, Torsten Schwede, Linda Thöny-Meyer

**Affiliations:** 1 Laboratory for Biointerfaces, Empa, Swiss Federal Laboratories for Materials Science and Technology, St Gallen 9014, Switzerland; 2 Biozentrum, University of Basel, Klingelbergstrasse 50/70, Basel 4056, Switzerland; 3 SIB Swiss Institute of Bioinformatics, Klingelbergstrasse 50/70, Basel 4056, Switzerland; 4 GlycoVaxyn AG, Schlieren 8952, Switzerland

**Keywords:** *N*-glycosylation, oligosaccharyltransferase, *Campylobacter jejuni*, PglB, directed evolution, protein modelling

## Abstract

Conjugate vaccines belong to the most efficient preventive measures against life-threatening bacterial infections. Functional expression of *N*-oligosaccharyltransferase (*N*-OST) PglB of *Campylobacter jejuni* in *Escherichia coli* enables a simplified production of glycoconjugate vaccines in prokaryotic cells. Polysaccharide antigens of pathogenic bacteria can be covalently coupled to immunogenic acceptor proteins bearing engineered glycosylation sites. Transfer efficiency of PglB*_Cj_* is low for certain heterologous polysaccharide substrates. In this study, we increased glycosylation rates for *Salmonella enterica* sv. Typhimurium LT2 O antigen (which lacks *N*-acetyl sugars) and *Staphylococcus aureus* CP5 polysaccharides by structure-guided engineering of PglB. A three-dimensional homology model of membrane-associated PglB*_Cj_*, docked to the natural *C. jejuni N*-glycan attached to the acceptor peptide, was used to identify potential sugar-interacting residues as targets for mutagenesis. Saturation mutagenesis of an active site residue yielded the enhancing mutation N311V, which facilitated fivefold to 11-fold increased *in vivo* glycosylation rates as determined by glycoprotein-specific ELISA. Further rounds of *in vitro* evolution led to a triple mutant S80R-Q287P-N311V enabling a yield improvement of *S. enterica* LT2 glycoconjugates by a factor of 16. Our results demonstrate that bacterial *N*-OST can be tailored to specific polysaccharide substrates by structure-guided protein engineering.

## 2. Introduction

**Figure 1.**
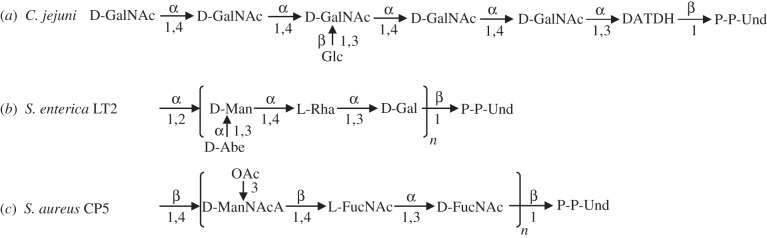
Structures of oligo- and polysaccharide substrates of PglB*_Cj_* analysed in this study. (*a*) *Campylobacter jejuni* heptasaccharide [4]; GalNAc, 2-*N*-acetylgalactosamine; Glc, glucose; DATDH, 2,4-diacetamido-2,4,6-trideoxyhexose; P-P-Und, undecaprenyl-pyrophosphate. (*b*) *Salmonella enterica* sv. Thypimurium LT2 *O*-antigen polysaccharide [5]; Man, mannose; Abe, abequose (3,6-deoxy-d-galactose); Rha, rhamnose; Gal, galactose. (*c*) *Staphylococcus aureus* capsular polysaccharide type 5 [6]; ManNAcA, 2-acetamido-2-deoxy-D-Mannuronic acid; OAc, *O*-acetyl modification; FucNAc, 2-*N*-acetylfucosamine.

N-linked glycosylation is a key posttranslational protein modification in eukaryotic cells. Bacterial *N*-oligosaccharyltransferases (*N*-OSTs) perform a similar enzymatic reaction in prokaryotic cells [1]. The protein glycosylation gene cluster of *Campylobacter jejuni*, including the *pglB* gene encoding a membrane-bound *N*-OST, can be expressed in the standard bacterial host *Escherichia coli*, resulting in glycosylation of a co-expressed periplasmic protein carrying at least one surface-exposed D/E-Y-N-X-S/T (Y, X ≠ P) glycosylation motif [2,3]. PglB*_Cj_* has an extraordinarily relaxed specificity for the lipid-linked polysaccharide substrate. Not only does it transfer the natural *C. jejuni* oligosaccharides (OSs) (figure 1*a*), but also O antigen lipopolysaccharide structures of numerous Gram-negative bacteria and capsular antigen polysaccharides of Gram-positive bacteria [7–11]. Furthermore, it is possible to combine this relaxed glycan specificity with the introduction of glycosylation site consensus sequences at specific positions of any desired protein for the production of custom glycoproteins in *E. coli* [12,13]. This technology is of particular value for the production of conjugate vaccines where surface polysaccharide antigens of bacterial pathogens are covalently bound to immunogenic protein carriers inducing long-lasting, strong humoural immune responses towards the polysaccharide of the pathogen [1,11]. Conventional conjugate vaccines of this kind produced by chemical coupling belong to the most effective and safest vaccines, and have been used in humans for over 30 years [14–18].

Although PglB*_Cj_* exhibits a relaxed specificity for the lipid-bound glycan substrate, polysaccharides missing a C2 *N*-acetyl group at the reducing end monosaccharide are transferred less efficiently to acceptor proteins than the natural *C. jejuni* OS or other C2 *N*-acetylated glycans. For example, in the case of polysaccharides with a hexose at the reducing end such as *Salmonella enterica* sv. Typhimurium LT2 (figure 1*b*) and *E. coli* capsular polysaccharide K30, glycosylation was not detectable [8]. The requirement of an acetamido group at the C2 position of the innermost sugar has been attributed due to critical hydrogen bonding to the *N*-OSTs catalytic site and/or stabilization of an oxonium reaction intermediate [8]. The glycan structures of natural N-linked bacterial glycoproteins and engineered glycoconjugates synthesized with PglB*_Cj_* almost exclusively contain *N*-acetyl substituted carbohydrates at the reducing end [4,7,9–12,19,20].

A common strategy to increase the activity of enzymes towards non-natural substrates is to perform directed evolution [21,22]. This technique consists of repetitive rounds of diversity generation at the DNA level combined with screening for mutants with a desired activity, resulting in a stepwise optimization of the enzyme [23]. Fully random approaches rely on techniques such as error-prone PCR and DNA shuffling. Large libraries are generated and screened using high-throughput assays in order to find improved variants [24,25]. Structure-guided strategies (in particular iterative active site saturation mutagenesis) considerably reduce the screening effort necessary for finding improved variants [26,27].

**Figure 2.**
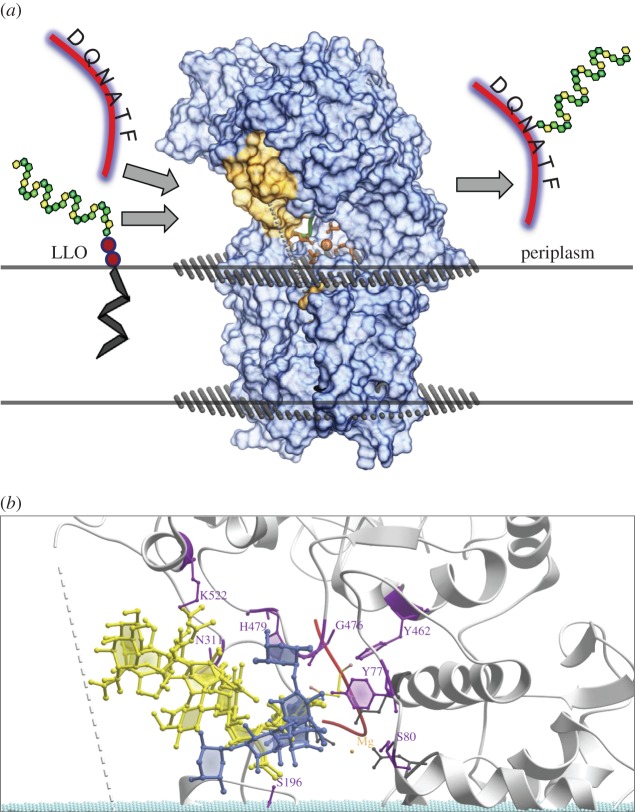
Modelling of OS structures interacting with PglB*_Cj_*. (*a*) Schematic of the envisioned glycosylation by PglB*_Cj_* in the periplasm and the overall structure of the homology model generated in this study. A lipid-linked oligosaccharide (LLO) is captured in the active site and the OS (repeating hexagon multimers) is covalently attached to the asparagine of the acceptor peptide (red tube). The pyrophosphate group is shown in red and the undecaprenyl moiety anchored in the lipid membrane as a black zig-zag line. The active site is composed of the orange residues shown in ball and stick mode and the Mg^2+^ ion (orange sphere). The exact sequence of steps from the delivery of the LLO to the release of the glycosylated substrate/protein is not known. (*b*) Close-up view of representative low energy conformations within the respective ensembles of structures of *C. jejuni* OS (yellow) and *S. enterica* LT2 repeating unit (blue) in the active site. The PglB*_Cj_* backbone structure is shown in grey (ribbon, surface) and the phosphate groups of the membrane as cyan-coloured balls. Residues in close proximity to the natural OS are depicted as magenta-coloured ball–stick representations. The strictly conserved residues acting as ligands for the divalent metal cofactor are shown as grey ball–stick representations. Broken line: approximate position of unstructured external loop EL5.

The recent elucidation of the crystal structure of *N*-OSTs of *Campylobacter lari* facilitates structure-guided engineering of PglB*_Cj_. Campylobacter lari* PglB exhibits 56% amino acid identity to PglB*_Cj_* and is also active when expressed in *E. coli* [28]. The crystal structure with bound synthetic D-Q-N-A-T-*p*-nitrophenylalanine acceptor peptide revealed that both the transmembrane and the soluble periplasmic domains of bacterial *N*-OSTs participate in substrate binding and catalysis [29] (see also figure 2*a*). The active site was found to be organized in two halves, one being responsible for protein binding and the other probably being involved in binding of the undecaprenyl-pyrophosphate-linked OS substrate, and in bond formation between the innermost sugar and the asparagine amide nitrogen of the acceptor peptide [29]. The large external loop EL5 of the transmembrane domain (residues S280 to D324 in *C. lari* PglB) separates both halves. Its C-terminal part was found to pin the acceptor peptide to the active site in the X-ray structure, forming a ‘porthole’ for the amido group of the acceptor asparagine, while no structural information could be obtained for the N-terminal part of EL5 [29]. Experimental evidence indicates that EL5 plays a central role in catalysis by undergoing extensive conformational changes in the course of substrate binding and product release [30,31]. A crystal structure of an *N*-OST with bound OS substrate is not yet available. Therefore, direct evidence for the interaction of specific active site residues with the OS and the undecaprenyl-pyrophosphate carrier is missing. Mutation of the conserved residue Y293 of the N-terminal part of EL5 abolished glycosylation, but not peptide binding [31], indicating an interaction with the OS substrate. Apart from Y293, the X-ray structure and mutational studies indicate that a loop of the periplasmic domain encompassing residues PglB*_Cl_* D481-G487, corresponding to PglB*_Cj_* D475-G481, may interact with bound OSs [29,32,33].

In this study, we attempted to evolve PglB*_Cj_* for enhanced transfer of heterologous polysaccharide substrates. Selection of residues for mutagenesis was guided by a PglB*_Cj_ in silico* model interacting with peptide–OS conjugates resembling the product state of the glycosylation reaction. Mutation of non-conserved, predicted sugar-interacting residues yielded improved variants facilitating increased production rates of glycoconjugate vaccines containing *S. enterica* LT2 and *S. aureus* CP5 polysaccharides (figure 1*c*).

## 3. Results

### 3.1. Modelling of PglB*_Cj_* and *in silico* oligosaccharide binding

To select potential OS interacting residues of PglB*_Cj_* as targets for mutagenesis, we constructed a structural model of PglB*_Cj_*. The experimental structure of *C. lari* PglB (PDBid 3RCE) was used as template for homology modelling. A simplified reaction scheme and the overall structure of PglB*_Cj_* is shown in figure 2*a*, including bound acceptor peptide, the metal cofactor and a phospholipid bilayer model. The model comprises coordinates for residues 1–713 of the wild-type PglB*_Cj_* sequence (PglBwt, UniProt: A8FMH8) except the C-terminal part of loop EL5 (residues 280–304), which is disordered in the template structure. In a next step, the natural *C. jejuni N*-glycan OS (figure 1*a*) and the first repeating unit of the heterologous *S. enterica* LT2 polysaccharide (figure 1*b*), respectively, were modelled by sampling their available conformational space within PglB*_Cj_*. The conformation of the enzyme–ligand complex in the model was assumed to be product-like (i.e. sampling was constrained by the covalent attachment of the OS to amide nitrogen of the asparagine residue of the acceptor peptide). Ensembles of predicted conformations of *C. jejuni* OS and the first repeating unit of *S. enterica* LT2 are shown in figure 2*b*. Overall, the relative orientation of the two OSs in the binding site and their interactions with the protein exhibited significant differences. Snapshots of the thermodynamically most favoured conformations are shown in electronic supplementary material, figure S1.

The *C. jejuni* OS exhibited a variety of interactions to itself and to the surrounding residues of wild-type PglB*_Cj_* mediated by *N*-acetyl moieties. By contrast, the LT2 OS moiety showed fewer carbohydrate protein interactions, probably due to the lack of *N*-acetyl substituents (figure 2*b*). This supports the hypothesis that low transfer efficiency of non-natural glycan substrates is caused by poor binding to the active site of PglB*_Cj_*.

To identify mutagenesis positions within the PglBwt sequence, we selected amino acid positions in the structural model with side chains located within 4Å distance to the ensemble of conformations of the *C. jejuni* OS. Residues Y77, S80, S196, N311, Y462, H479 and K522 of PglB*_Cj_* matched this criterion (figure 2*b*). In the case of G476 and G477, the carbonyl oxygen atoms of the polypeptide backbone were predicted to be within 4Å distance to the innermost sugar (figure 2*b*).

### 3.2. Mutagenesis of predicted sugar-interacting residues

To check if our strategy was meaningful, we mutated the selected amino acid positions and analysed the effects on glycosylation efficiency. In the first round of experiments, activity towards the LT2 substrate was measured, using an ELISA assay that was specifically developed for this purpose [33].

**Figure 3.**
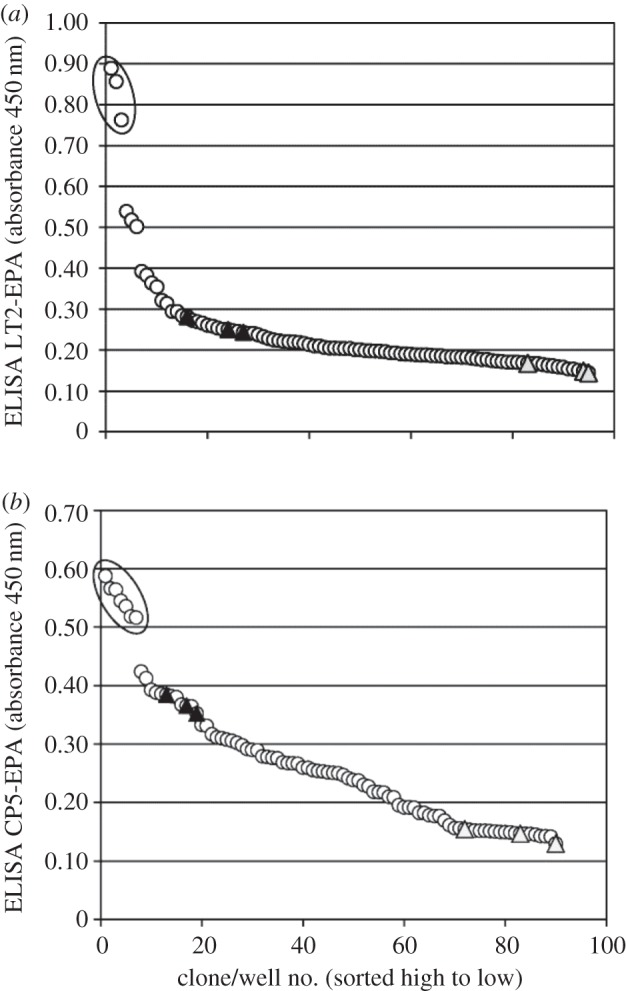
Saturation mutagenesis of PglB*_Cj_* residue N311, library screening by sandwich ELISA. (*a*) Host strain and detection antibodies for *S. enterica* LT2 polysaccharides. (*b*) Host strain and detection antibodies for *S. aureus* CP5 polysaccharides. Open circles, library clones; filled triangles, positive control clones-expressing wild-type PglB (pGVXN1413); shaded triangles, negative control clones-expressing inactive PglB_mut_ (pGVXN408). Sequenced clones are marked by an ellipsoid.

The first position tested was N311. In the structural model of PglB*_Cj_* with interacting *C. jejuni* OS, the amide group of N311 formed a hydrogen bond with the C6 hydroxyl group of the second (counted from the reducing end bacillosamine) monosaccharide (i.e. GalNAc; electronic supplementary material, figure S1). N311 was fully randomized to all natural amino acids. For glycosylation tests, we used *S. enterica* serovar Typhimurium strain LT2 expressing the O : 4 factor O antigen and a non-toxic form of *Pseudomonas aeruginosa* exotoxin (EPA) from a plasmid. The plasmid library with the randomized N311 position was transformed into the *Salmonella* test strain and screened with a glycoprotein-specific sandwich ELISA (see Material and methods section). Three clones showed significantly improved glycosylation efficiency compared with wild-type PglB (figure 3*a*). In all of them, residue N311 was mutated to valine.

These results confirmed the utility of our approach and experimental set-up. However, it was still unclear if the discovered improvement was due to a specificity alteration for the LT2 substrate or a generic activity enhancement. To address this question, we tested the same fully randomized N311 library regarding the transfer of different glycan, namely the *S. aureus* capsular polysaccharide Type 5 (CP5, figure 1*c*), which we have established previously as substrate for EPA glycosylation in *E. coli* [11,33]. Seven clones with an improved glycosylation efficiency compared with wild-type PglB were identified when measuring CP5-EPA productivity by ELISA (figure 3*b*). Six of them harboured the amino acid substitution N311V (codons GTG and GTT) and one exhibited mutation N311I (codon ATT).

Amino acid changes often have detrimental effects. Considering all clones yielding at least 10% of wild-type ELISA signals (background-corrected), the majority of N311 variants was active (≈80%). This indicates that N311 is highly mutation tolerant, which is in agreement with considerable variability at this position in homologous *N*-OSTs sequences (electronic supplementary material, figure S2).

In summary, these experiments led to the identification of N311 as a position with high impact on glycosylation rates. The effect was only partially substrate dependent. However, as the experimental system was based on a high-copy-number plasmid library and on glycosylation during growth in deep well plates, we analysed the activities of the N311V mutant, expressed from a different vector, in replicate shake flask cultures.

### 3.3. Effect of N311V on glycoprotein formation rates

**Figure 4.**
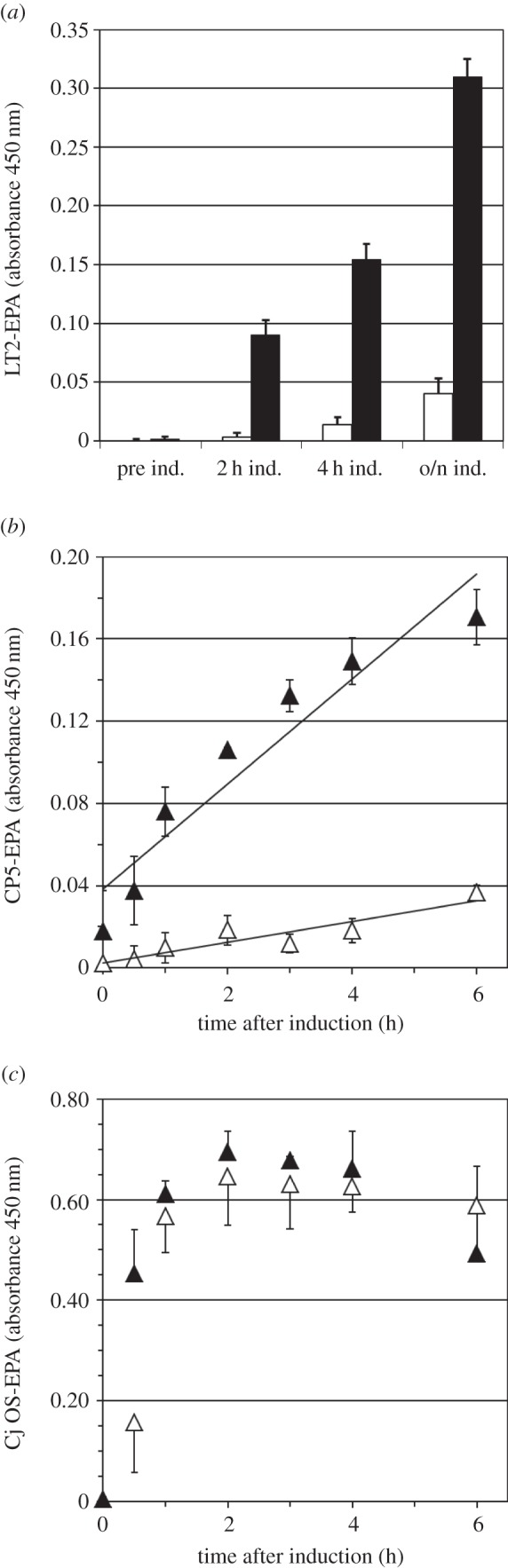
Effect of amino acid substitution PglB*_Cj_* N311V on glycosylation of EPA with two heterologous polysaccharides and natural OSs. Open symbols, wild-type PglB (pGVXN970); closed symbols, PglB N311V (pGVXN1217). (*a*) Host strain and detection antibodies for *S. enterica* sv. Thyphimurium LT2 polysaccharides, (*b*) host strain and detection antibodies for *S. aureus* CP5 polysaccharides, and (*c*) host strain and detection antibodies for *C. jejuni* OSs. Background-corrected ELISA signals for biomass-normalized periplasmic extracts from shake flask cultures, average values and standard deviations of *n* = 3 biological replicates.

The identified beneficial mutation N311V was introduced into wild-type PglB*_Cj_*, expressed from the low-copy-number vector pEXT21 by site-directed mutagenesis. Wild-type (pGVXN970) and mutant (pGVXN1217) plasmids were transformed into the same expression cell lines as above and tested for glycosylation. Productivity was measured using a time-resolved ELISA analysis (figure 4*a*). The mutant *N*-OSTs yielded eightfold more LT2-EPA after overnight induction (figure 4*a*). The improvement factors after 2 and 4 h of induction were 22- and 11-fold, respectively. The initial rate of CP5-EPA formation was increased by a factor of 5.1 (figure 4*b*). Although N311 is conserved in PglB sequences of numerous *Campylobacter* species (electronic supplementary material, figure S2), no significant effect was found for *in vivo* glycosylation of EPA with the natural *C. jejuni* OS substrate (figure 4*c*). The increase in ELISA signals over time and the beneficial or neutral effect of N311V corresponded to western blot results for exemplary periplasmic protein samples (electronic supplementary material, figure S3).

In principle, increased *in vivo* activities of enzyme variants can arise from either increased catalytic efficiency or higher levels of functional protein. To analyse the effect of mutation N311V on expression of PglB, we constructed wild-type and N311V variants fused to a C-terminal haemagglutinin (HA) peptide tag. This allowed us to follow expression levels of PglB over time. PglB-HA-specific bands in biomass-normalized whole cell protein samples originating from mutants were less intense and more variable than those from wild-type PglB (electronic supplementary material, figure S4*a*). Degradation products corresponding in size to the C-terminal periplasmic domain appeared after induction, indicating a destabilizing effect of the mutation. Nevertheless, CP5-EPA production followed by ELISA was again significantly increased in cells expressing PglB*_Cj_* N311V (electronic supplementary material, figure S3*b*). Thus, we concluded that the PglB*_Cj_* N311V variant exhibits improved activity due to a mechanism independent of protein stability.

Finally, we studied the effect of mutation N311V on glycosylation of a different acceptor protein with LT2 polysaccharides. Mutant and wild-type plasmids were transformed into *S. enterica* SGSC228 that co-expressed an engineered variant of *C. jejuni* AcrA (40 kDa) with three glycosylation sites. Using anti-*Salmonella* O : 5 antiserum, much stronger bands in the size range of 60–170 kDa were detected in periplasmic extracts of the strain expressing PglB*_Cj_* N311V compared with strains expressing either wild-type or inactive PglB*_Cj_* (electronic supplementary material, figure S5). This indicates that the improvement is glycan-specific and does not depend on the type of carrier protein.

### 3.4. Further rounds of mutagenesis and screening

In directed evolution, repetitive rounds of mutagenesis and screening led to additive improvements of the desired property [27]. Following the principle of iterative saturation mutagenesis, PglB*_Cj_* N311V was used as template for randomization of other positions that had been selected with the help of the structural model. Y77 and S80 were found to be highly mutation tolerant. When screened for LT2-EPA production, more than 70% of all clones were active (table 1). PglB*_Cj_* Y77 and S80 are not conserved in bacterial *N*-OSTs homologues (data not shown). The 10 clones with the highest ELISA signals were sequenced. Y77 was changed to diverse amino acids, with a bias towards residues with basic side chains (table 1). In the NNK library randomizing S80, variant S80R was dominating in the top-performing clones (table 1).

**Table 1.** Mutation tolerance of PglB*_Cj_* residues mutated in second round saturation mutagenesis libraries and amino acid substitutions identified in the 10 clones with the highest LT2-EPA ELISA signals. Clones were counted as active when background-corrected ELISA signals reached more than 10% of the average value of N311V control wells.

PglB*_Cj_* residue	fraction of active clones in NNK library (%)	mutations identified in top 10 clones
Y77	86	Y77H (2x) Y77T Y77W Y77R Y77K (2x) Y77A Y77G
S80	81	S80R (8x) S80H
Q287	65	Q287P (4x) Q287K (2x) Q287R
L288	61	L288M (2x) L288F L288I L288I
K289	78	K289R (4x) K289N (2x) K289Q (2x)
F290	16	none (all wt)
Y291	3.9	none (all wt)
R294	22	R294K

The PglB*_Cj_* Q_287_LKFYxxR_294_ motif within the N-terminal part of EL5 is highly conserved in PglB sequences of *Campylobacter* species, but not in *N*-OSTs of more distantly related species (electronic supplementary material, figure S2). Due to the observation that the innermost two sugar subunits of N-linked glycans of *Campylobacter* species are similar (1st 2,4-diacetamido-2,4,6-trideoxyhexose, 2nd *N*-acetyl-hexosamine) [4], we hypothesized that residues of the *Campylobacter*-specific Q_287_LKFYxxR_294_ motif may influence OS specificity. Saturation mutagenesis libraries were generated at these positions with improved variant N311V as template. When screened in the host strain for LT2-EPA production, a clear difference was observed for the first and second part of the motif (table 1). While saturation mutagenesis of Q287, L288 and K289 yielded 60–80% active clones, the adjacent residues F290, Y291 and R294 were highly mutation-sensitive. The 10 top-performing clones of the Q287, L288 and K289 libraries exhibited non-random amino acid substitutions (table 1). Proline and the positively charged amino acids lysine and arginine were present at position Q287. At L288, alternative hydrophobic residues (M, I, F or C) were found exclusively. A bias for residues with either amide (Q, N) or positively charged side chains (R) was observed at position K289 (table 1).

**Figure 5.**
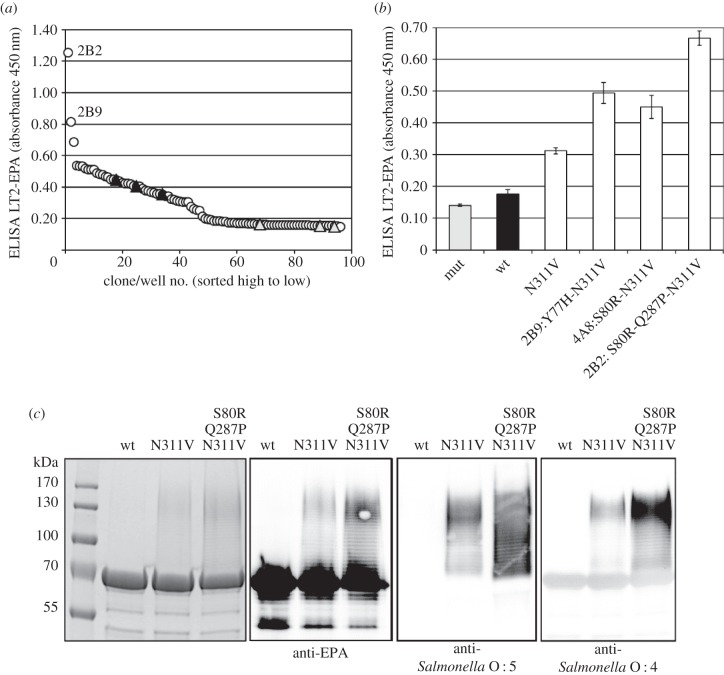
Shuffling of neutral and slightly beneficial mutations of PglB*_Cj_*. (*a*) Screening results for a representative 96-well library; open circles, library clones; filled triangles, PglB N311V (template plasmid pGVXN1418); shaded triangles, inactive PglBmut (pGVXN408). (*b*) Verification of improvements after retransformation, average values and standard deviations for *n* = 3 replicate clones/wells per variant plasmid; wt, pGVXN1413; N311V, pGVXN1418. (*c*) SDS-PAGE and western blot analysis of Ni-NTA affinity purified proteins produced with either wild-type PglB (pGVXN970), PglB N311V (pGVXN1217) or PglB S80R-Q287P-N311V (library clone 2B2) in shake flasks; similar loading volumes, total protein concentration (A280) was adjusted to 2 mg ml^−1^. Theoretical molecular weight of unglycosylated EPA-6H: 69.4 kDa.

In a final step, the neutral and slightly beneficial mutations occurring at Y77, S80, Q287, L288 and K289 were shuffled. When 720 clones of this library were screened for LT2-EPA production, numerous positive outliers were identified as shown exemplarily for one 96-well screening plate in figure 5*a*. Clones with at least 2.5-fold increased ELISA signals compared with the average signal of template control clones on the same plate were sequenced (*n* = 14). S80R was detected in 79% (*n* = 11), Q287P in 43% (*n* = 6) and Y77H in 29% (*n* = 4) of these clones, respectively. The double mutants Y77H-N311V and S80R-N311V were found two and four times, respectively. Q287P occurred only in combination with either Y77H or S80R. Y77S, L288I, L288F, K289R and K289Q were found once or twice in combination with the more frequently observed mutations, indicating that these amino acid substitutions were neutral.

Representative improved variant plasmids were retransformed into the LT2-EPA expression strain and rescreened in triplicate DWP mini cultures (figure 5*b*). A significant additive enhancing effect was verified for both Y77H (2.1-fold to N311V) and S80R (1.8-fold to N311V). When combined with S80R-N311V, Q287P led to a further increase in EPA-LT2 ELISA signals by a factor of 1.7, resulting in a total improvement factor of 15 relative to wild-type PglB in overnight induced DWP cultures (figure 5*b*). An additive increase of glycoprotein band intensity resulting from single and triple mutations was also observed for hexahistidine-tagged EPA purified by affinity chromatography from shake flask cultures (figure 5*c*). Glycoforms hybridized with antibodies specific for *Salmonella* serotypes O : 4 and O : 5, detecting the branching abequose sugar within the LT2 polysaccharide [5] and its *O*-acetylated form, respectively [34]. The mean intensity of LT2-EPA glycoform bands (more than 80 kDa) detected on the Coomassie-stained SDS polyacrylamide gel was analysed with image quantification software. Compared with wild-type PglB*_Cj_*, background-corrected Grey values were increased by factors of 9 and 16 for variants N311V and S80R-Q287P-N311V, respectively.

## 4. Discussion

Directed evolution is a powerful tool for generating improved enzymes enhancing biotechnological production processes [35]. Using previously established techniques for the screening of *N*-OSTs mutant libraries [33], we were able to demonstrate that PglB of *C. jejuni* can be evolved for enhanced transfer of heterologous polysaccharide substrates. Others have shown before that this enzyme can be engineered for enhanced glycosylation of non-canonical acceptor peptide sequences [36]. Protein glycosylation in recombinant *E. coli* bears high potential for a simplified production of high-quality conjugate vaccines, and engineered PglB*_Cj_* variants are expected to be key components of efficient, economically viable processes.

The key to success in engineering of PglB*_Cj_* in this study was the structure-guided selection of mutated residues. In order to identify potential OS-interacting residues, the natural *C. jejuni* heptasaccharide was modelled *in silico* in a product-like state, with the substrate covalently bound to the nitrogen atom of an acceptor peptide in a homology model of PglB*_Cj_* (analogous to the *C. lari* structure 3RCE). Whether this model accurately resembles the situation just before product release is not known, because any potential contributions of the C-terminal part of EL5 are not represented in the homology model. EL5 probably undergoes extensive rearrangements in the course of N-linked glycosylation [29,30].

Specific amino acid substitutions at the potential carbohydrate-interacting active site residues N311, Y77 and S80 had a positive effect on glycosylation of EPA with LT2. Enhancing mutations were obtained exclusively at mutation-tolerant PglB*_Cj_* residues that exhibited considerable sequence variability in bacterial *N*-OSTs homologues. In many successful structure-guided protein engineering studies, mutagenized active site positions were also characterized by a moderate to high degree of variability in phylogenetic alignments [37,38].

Wild-type residue N311 interacts with the natural *C. jejuni* OS according to our stuctural model. A change to valine at this position clearly increases transfer rates for several heterologous PS substrates. The exact mechanism of the beneficial effect of this mutation is unknown. It might confer additional flexibility to the C-terminal part of EL5, enabling the active site to better accommodate saccharide structures which differ from *C. jejuni* OS. Enhanced flexibility of the enzyme could increase susceptibility to degradation, as was observed in one of our experiments. Another explanation would be that a switch to valine at N311 renders the active site more hydrophobic, which in turn might force some PS substrates into a more favourable conformation for binding and transfer.

A change to histidine at position Y77 also increases LT2-EPA formation when combined with N311V. Apart from a potential stability-enhancing effect, this could be related to a change of the hydrogen bond network, resulting in an enhanced binding of the two innermost sugars of LT2. Likewise, the enhancing effect of mutation S80R might be due to either increased stability of PglB or novel hydrogen bond interactions facilitated by the longer side chain of arginine with its terminal amino group. The side chains of histidine and arginine are often hydrogen bond donors and acceptors for the substrate in oligohexose-binding enzymes (e.g. in maltodextrin phosphorylase [39]). Alternatively, and affecting the initial phase of the glycosyl transfer, the switch to the positively charged arginine at position S80 could alter the interaction with the (negatively charged) phosphate group of the undecaprenyl-pyrophosphate carrier, thereby enhancing the transfer of LT2 polysaccharides. The reason for the observed enhancing effect of Q287P (in combination with S80R-N311V) could be a stabilizing effect as well as a glycosylation-promoting change in the preferred conformations of EL5. Proline introduces rigidity in this part of EL5, thereby potentially affecting interactions with the LT2 saccharide. Both the enhancing effect of Q287P on LT2-EPA formation and the mutation sensitivity of PglB*_Cj_* Y291 (Tyr294 in PglB*_Cl_*) provide further evidence for an involvement of the N-terminal region of EL5 in OS binding [31].

The inner structure of precursor glycans transferred by *N*-OSTs to eukaryotic glycoproteins almost exclusively consists of Glc_3_Man_9_GlcNAc_2_ tetradecasaccharides [40]. The *N*-acetylglucosamine residue at the reducing end is covalently linked to the nitrogen atom of the asparagine in the protein glycosylation site. Experiments with unnatural dolichol-linked disaccharide analogues led to the conclusion that eukaryotic *N*-OSTs require an acetamido group at the C2 position of the innermost sugar, which allows critical hydrogen bond interactions with the active site [41]. Similarly, PglB*_Cj_* was found to predominantly transfer oligo- and polysaccharides with C2-acetamido substituted sugars. However, there is an interesting exception to this rule: PglB*_Cj_* catalysed N-linked glycosylation of AcrA with *Burkkolderia pseudomallei O*-polysaccharides that bear an *O*-acetylated deoxytalose at the reducing end [42]. Together with the latter study, our findings, demonstrating N-linked glycosylation of EPA and AcrA with *Salmonella* LT2 *O*-polysaccharides, suggest that the C2 *N*-acetyl substitution is not strictly required for glycan transfer by bacterial *N*-OSTs. This questions the importance of the previously proposed catalytic intermediate involving an *N*-acetyl cyclic transition state (oxonium intermediate) [8] for N-linked glycosylation in bacterial cells.

## 5. Material and methods

### 5.1. Protein modelling

A set of homology models of *C. jejuni* PglB was generated for the wild-type sequence (UniProt: A8FMH8, NCBI Reference sequence NC_009839.1, GI: 157415386) using a number of established homology modelling servers [43–46]. The expected quality of the models was estimated using QMEAN [47] and QMEANBrane [48]. A model generated with the HHpredB method [49] with most favourable QMEAN score was selected. The Mg^2+^ ion and the acceptor peptide were modelled in analogy to the *C. lari* structure (PDBid 3RCE). To visualize the orientation of the protein in the cytoplasmic membrane, a phospholipid bilayer model was derived from the OPM database entry for *C. lari* [50]. The three-dimensional coordinates of the PglB*_Cj_* model have been deposited at http://www.modelarchive.org under doi:10.5452/ma-azlj1.

The natural heptasaccharide ligand of *C. jejuni* and the first repeating unit of the *S. enterica* LT2 polysaccharides were parametrized using EPIK [51], accounting for charges and tautomers, and placed into the model. Each substrate was then conformationally sampled with the ‘mixed torsional/low mode sampling’/‘MCMM’ protocols available in MacroModel v. 9.9–10.2 (Schrödinger). The saccharide was defined as freely moving substructure; position constraints have been placed on all protein backbone atoms and side chain atoms outside the putative OS-binding site (more than 6Å apart from the OS). Distance constraints on the covalent bond between the first saccharide unit (N-C1) and the ASP-N (ND) were set at 1000 kJ mol^−1^Å^2^. The OPLS2005 force field including a full atom water model was used, and the first conformational sampling performed with 1000 steps and a 35 kcal mol^−1^ window to screen possible conformations, and refined with a similar protocol employing a more narrow energy window of 21 kJ mol^−1^.

### 5.2. Bacterial strains and plasmids

Bacterial strains and plasmids used in this study are described in electronic supplementary material, table S1. *Escherichia coli* W3110 *waaL* was used as host strain for production of *C. jejuni* OS-EPA-glycoconjugates and CP5-EPA in *in vivo* glycosylation experiments. *Salmonella enterica* sv. Typhimurium SGSC228, which produces LT2 polysaccharides and lacks a functional *waaL* gene, was used for production of LT2-EPA. Ultra-competent *E. coli* cells were used for initial transformation of plasmid libraries. *Escherichia coli* DH5α was used as standard host for plasmid production and storage. Appropriate antibiotics were added to all growth media to ensure plasmid maintenance (Ampicillin, Amp: 100 mg l^−1^, Chloramphenicol, Cm: 10 mg l^−1^, Kanamycin, Kan: 30 mg l^−1^, Spectimomycin, Sp: 80 mg l^−1^, Tetracyclin, Tet: 20 mg l^−1^).

Codon-optimized PglB in a high-copy-number cloning vector was obtained from a gene synthesis service company (GenScript, Piscataway, USA). The construction of template plasmids for *pglB* libraries is described in the electronic supplementary material. Low-copy-number, pEXT21-derived plasmids for expression of codon-optimized wild-type PglB and PglB N311V were obtained from GenScript.

### 5.3. Mutagenesis methods

Mutagenic primers and sequencing services were obtained from Microsynth (Balgach, Switzerland). PglB libraries were constructed by QuikChange using pGVXN1413, pGVXN1418 or pGVXN1930 as template. The sequences of oligonucleotides used for library construction and further experimental details are given in the electronic supplementary material. Plasmid libraries were produced by re-suspending and mixing of at least 1000 colonies (5000 colonies for the shuffled library) from transformations of ultra-competent *E. coli* in phosphate-buffered saline (PBS), followed by plasmid purification with a standard mini-prep kit. Successful randomization of desired residues was verified by sequencing of random clones and of the final plasmid library. Only libraries with less than 20% of wild-type clones were used for screening. Plasmid libraries were transformed into *E. coli* and *S. enterica* expression strains using standard electroporation procedures.

### 5.4. Screening of mutant libraries

Mutant libraries and individual variant plasmids were screened in 96 deep-well plates as described previously [33], except that the concentration of IPTG added at induction was reduced to 30 µM in order to reduce inclusion body formation. PglB variant plasmids were isolated from expression strains by retransformation of plasmid preps in chemically competent *E. coli* DH5α and selection for Kanamycin resistance only. Mutations were characterized by Sanger sequencing of purified plasmids, employing two overlapping reads. Chemically or electrocompetent *S. enteric*a SGSG228 (pGVXN150) and *E. coli* St1717 (pGVXN150, pGVXN393) were used as host strains for LT2-EPA and CP5-EPA DWP-ELISA screenings, respectively.

### 5.5. Shake flask experiments

Host strains for LT2-EPA and CP5-EPA production in shake flask experiments were similar to DWP experiments. *Escherichia coli* W3110 *waaL* (pACYC(*pgl*_mut_), pGVXN150) was used as host strain for *Cj* OS-EPA production. The kinetics of glycoprotein formation were recorded by preparing biomass-normalized periplasmic protein extracts at regular intervals after induction, followed by sandwich ELISA. Triplicate preculture tubes with LB medium (5 g l^−1^ yeast extract, 10 g l^−1^ and 5 g l^−1^ NaCl) were inoculated with individual single colonies from fresh streak-out or transformation plates and incubated overnight at 37°C and 160 r.p.m. Triplicate Erlenmeyer flasks with 50% v/v of LB-M9 medium (5 g l^−1^ yeast extract, 10 g l^−1^ tryptone, 12.8 g l^−1^ Na_2_HPO_4_ · 7H_2_O, 3.0 g l^−1^ KH_2_PO_4_, 0.5 g l^−1^ NaCl, 1.0 g l^−1^ NH_4_Cl, 2 mM MgSO_4_ · 7H_2_O and 0.1 mM CaCl_2_) were inoculated 1 : 50 from tube pre-cultures and incubated at 37°C and 160 r.p.m. At an OD_600_ of 0.5, 1 mM IPTG and 4 g l^−1^ l-arabinose were added for induction and stirrer speed was reduced to 100 r.p.m. In the case of the *Salmonella* host strain, EPA/EPA-LT2 degradation was observed in LB-M9 shake flask cultures after overnight induction. Degradation could be prevented by using a high-strength complex medium (2YT, 10 g l^−1^ yeast extract, 14 g l^−1^ tryptone, M9 salts), reducing OD_600_ at induction to 0.3–0.4 and switching to static incubation (i.e. fully anaerobic growth) after induction.

Periplasmic extracts were prepared as described previously [12]. Extracts were diluted 1000- to 20 000-fold in PBS with 1% w/v dry milk and analysed by sandwich ELISA. Only dilutions yielding non-saturated ELISA signals (absorbance at 450 nm below 1.0) were used for data analysis. For purification of hexahistidine-tagged proteins, periplasmic extracts of triplicate overnight shake flask cultures were pooled and Ni-affinity chromatography was performed according to standard protocols using HiTrap FF columns (GE Healthcare). SDS-PAGE and Coomassie staining were carried out using standard methods. The combined intensity of LT2-EPA glycoform bands on Coomassie-stained SDS polyacrylamide gels was quantified with the software ImageJ (imagej.nih.gov) using background-corrected mean Grey values.

### 5.6. ELISA and western blot analysis

Extracts of periplasmic proteins were diluted appropriately and analysed by sandwich ELISA in 96-well plates as described before [33]. The capture antibody for all ELISA analyses was protein G purified goat-anti-EPA antiserum [33]. Rabbit anti-*S. aureus* CP5 [33], rabbit anti-*Salmonella* O : 5/O : 4 (Staten Serum Institute, Denmark) and rabbit anti-*C. jejuni* [2] were used for detection of EPA-linked oligo- and polysaccharides. Horseradish peroxidase (HRP)-coupled goat-anti-rabbit IgG (Bio-Rad, Reinach, Switzerland) and Ultra-TMB substrate (Thermo-Scientific/Pierce) were used for ELISA development. The HRP reaction was stopped by addition of 2M H_2_SO_4_ and absorbance at 450 nm (ELISA signal) was measured against air with a plate reader. Appropriate development times were chosen so that signal saturation could be avoided (maximal abs. 450 nm ≤ 1.0). Signals were background-corrected by subtracting average absorbance values of samples derived from isogenic control strains expressing PglB_mut_. Western blot analysis of periplasmic and total cell proteins was performed as described previously [33]. EPA and PglB-HA were detected by rabbit anti-EPA (Sigma-Aldrich, Buchs, Switzerland) and rabbit anti HA (Sigma-Aldrich) primary antibodies, respectively. Oligo- and polysaccharides conjugated to EPA were detected with the same antibodies as used for ELISA.

## Supplementary Material

Supplementary_figures.docx

## Supplementary Material

Experimental_details.docx
