# Supplementary material for: Increased efficiency of Campylobacter jejuni N-oligosaccharyltransferase PglB by structure-guided engineering
Source: Open Biol. 2015 Apr 1;5(4):140227. doi: 10.1098/rsob.140227 (PMC4422122; doi:10.1098/rsob.140227)
Supplement: Supplementary_figures.docx [file rsob140227supp1.docx]

**Supplementary Figures**

**Suppl. Fig. S1.** Representative conformations of (*a*) *C. jejuni* OS and (*b*) *S. enterica* LT2 PS repeating unit in the PglB*_Cj_* model. Predicted hydrogen bond interactions between saccharide subunits and protein are shown as green dotted lines. Oligosaccharides are depicted as yellow or blue ball-stick representations with oxygen atoms in red. The PglB_Cj_ backbone structure is shown in grey (ribbon, surface) and the phosphate groups of the membrane as cyan-colored balls. Residues in close proximity to the natural OS are depicted as magenta-coloured ball-stick representations.

*
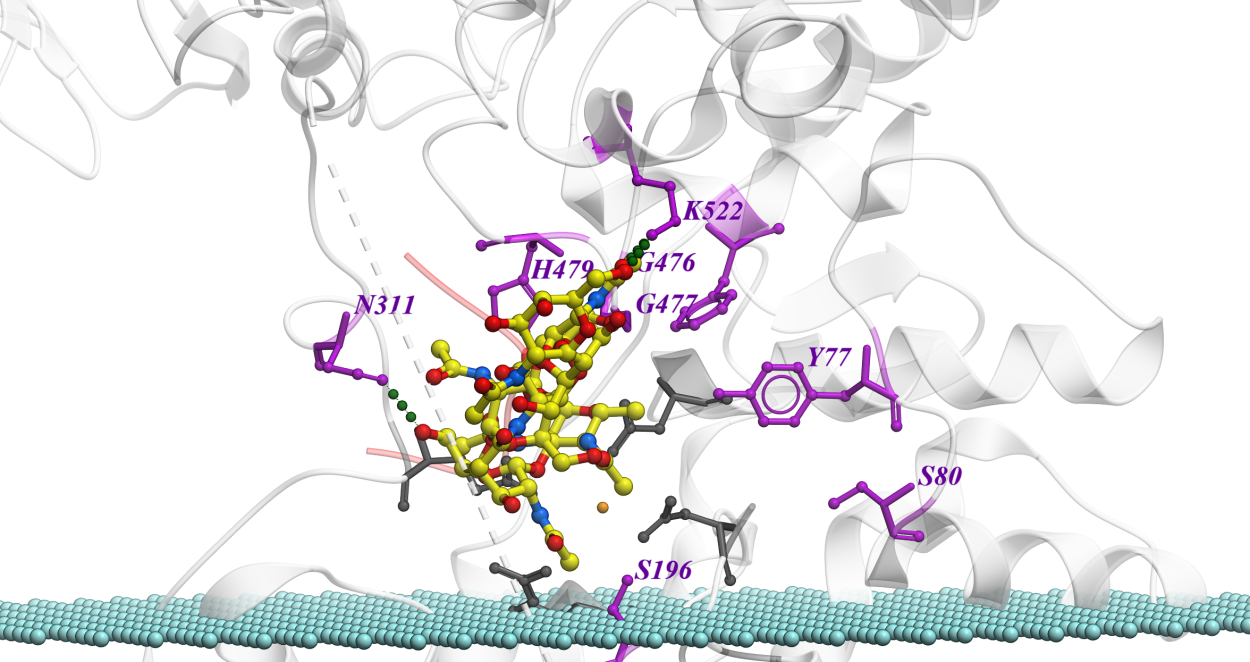
(a)*

*
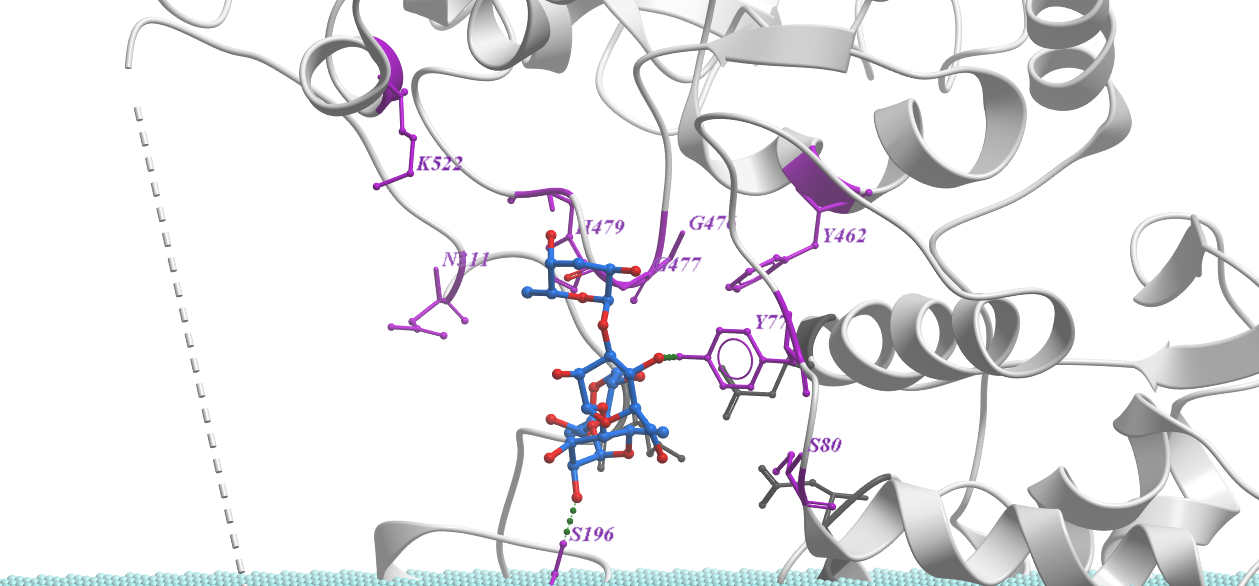
(b)* **Suppl. Fig. S2. Alignment of bacterial N-OST homologues in the vicinity of PglB*_Cj_* N311 (EL5 region).** PglB*_Cj_* was used as search template for Protein BLAST and non-redundant sequences were aligned with the MegAlign^TM^ program using the ClustalW algorithm (DNASTAR, Madison, WI, USA). PglB*_Cj_* residues conserved in sequences of other species are shaded, positions mutated in this study are boxed. PglB*_Cj_* E316 (*): Strictly conserved residue which is a ligand of both the divalent metal cofactor and the asparagine amine of the acceptor peptide in the PglB*_Cl_* crystal structure PDBid 3RCE.

*****

320

310

300

290

*Cj* 280

285


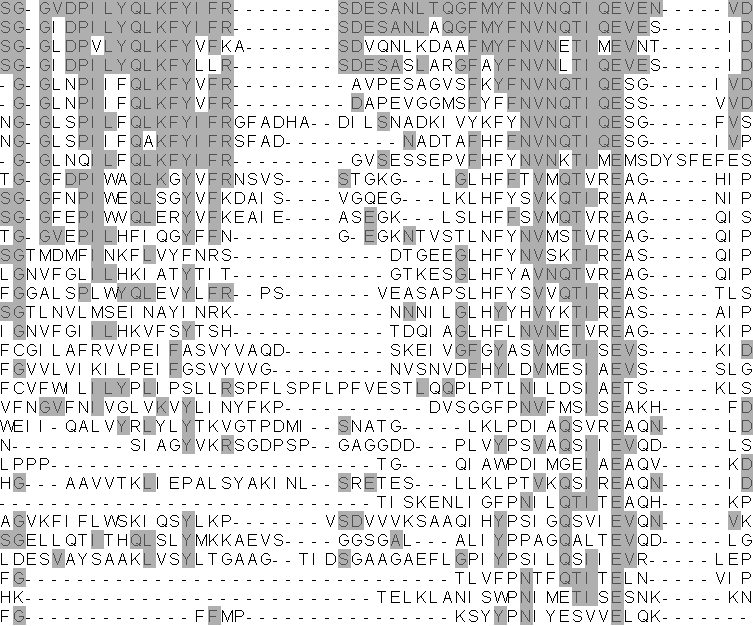


*C. jejuni*

*C. coli*

*C. lari*

*C. upsaliensis*

*C. curvus*

*C. concisus*

*C. hominis*

*C. gracilis*

*C. showae*

*S.autotrophica*

*S. denitrificans*

*S. deleyianum*

*S. kujiense*

*N. profundicola*

*S. sp. NBC37-1*

*W. succinogenes*

*C. mediatlanticus*

*N. sp. SB155-2*

*H. pullorum*

*H. canadensis*

*H. winghamensis*

*D. thermolithotr.*

*D. baculatum*

*D. vulgaris*

*D. alkaliphilus*

*D. retbaense*

*D. desulfuricans*

*D. salexigenes*

*D. piger*

*D. aespoeensis*

*Cand. P. marinum*

*C. nitroreducens*

*M. fervidus*

**Suppl. Fig. S3. Effect of mutation PglB*_Cj_* N311V on glycoprotein formation in shake flask analyzed by Western blot.** (*a*) LT2-EPA, host strain *S. enterica* SGSC228 (pGVXN150); (*b*) CP5-EPA, host strain *E. coli* St1717 (pGVXN150, pGVXN393); (*c*) EPA-*C. jejuni* OS, host strain *E. coli* CLM24 (pACYC(*pgl_mut_*), pGVXN150). Same experiments as shown in Fig. 4, biomass-normalized periplasmic extracts, similar loading volumes, samples of one shake flask culture per variant. Wild-type PglB: pGVXN970, PglB N311V: pGVXN1217. Theoretical molecular mass of unglycosylated EPA-6H: 69.4 kDa.

(*a*)

PglB N311V

wild-type PglB

time after induction: 0h 2h 4h o/n 0h 2h 4h o/n


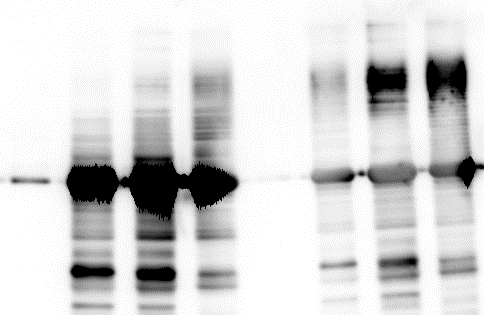


kDa

170

130

100

70

55

40

anti-EPA


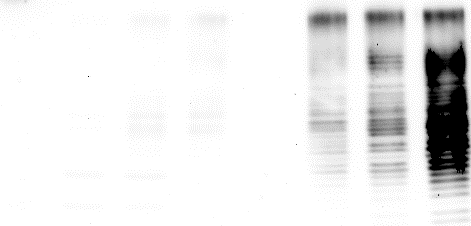


kDa

170

130

100

70

55

anti-*Salmonella*
O:5 (LT2)

(*b*)

PglB N311V

wild-type PglB

time after induction: 0h 1h 3h 6h 0h 1h 3h 6h


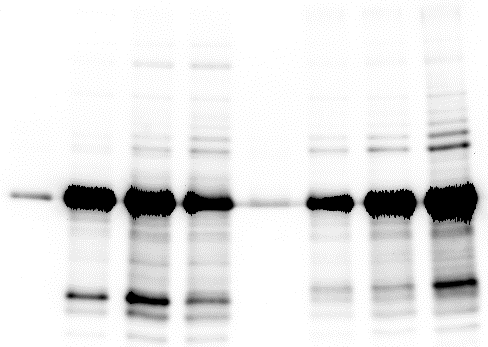


anti-EPA

kDa

170

130

100

70

55

40

kDa

170

130

100

70

55

40


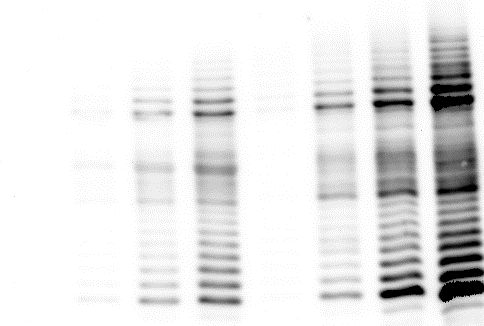


anti-*S. aureus* CP5

(*c*)


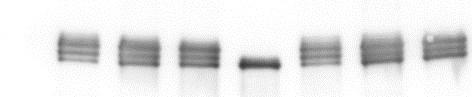


host

PglB N311V

wild-type PglB

time after induction: 0h 1h 3h 6h 6h 1h 3h 6h

anti-EPA


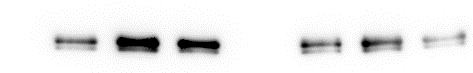


anti-*C. jejuni* OS

**Suppl. Fig. S4. Effect of N311V on expression of HA-tagged PglB and CP5-EPA formation in shake flask.** (*a*) Anti-HA Western blot analysis of PglB expression. Cell pellets of three replicate shake flask cultures per variant were resuspended to OD_600_ = 20 in SDS-PAGE sample buffer and denatured for 1h at 60°C. (*b*) Time course of CP5-EPA formation analyzed by sandwich ELISA of OD_600_-normalized periplasmic extracts. Open symbols: wild-type PglB-HA, closed symbols: PglB-HA N311V. Average values and standard deviations for n = 3 replicate cultures, absorbance values were corrected for PglBmut background. HA-tagged PglB and PglB N311V were expressed from plasmids pGVXN1929 and pGVXN1930, respectively, in host strain *E. coli* St1717 (pGVXN150, pGVXN393). Arrows: 1 full-length PglB, 2 degradation product of PglB.

(*a*)

6 h after induction

3 h after induction

PglB-HA
N311V

PglB-HA
N311V

wild type PglB-HA

host strain

wild type PglB-HA

host
strain

kDa

100

70

55

40

35

kDa

100

70

55

40

35


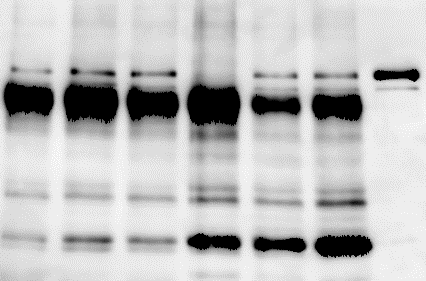

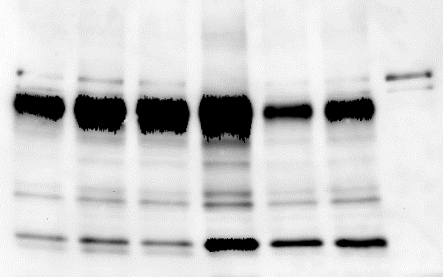


**1**

**2**

Anti-HA

Anti-HA

(*b*)


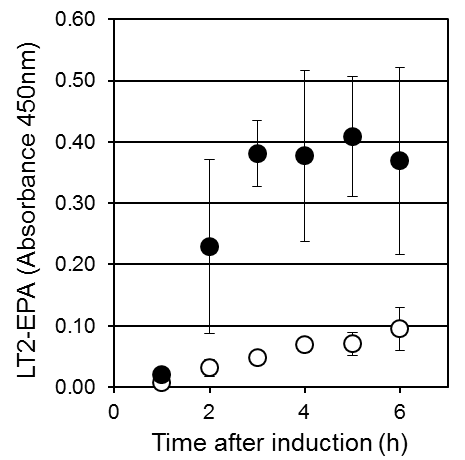


**Suppl. Fig. S5. Effect of PglB mutation N311V on glycosylation of AcrA with *Salmonella* *enterica* LT2 polysaccharides.** (*a*) Anti-*Salmonella* O:5 Western blot (primary antibody: rabbit anti-*Salmonella* O:5, Staten Serum Institute; secondary antibody goat anti-rabbit IgG-HRP, Biorad), (*b*) anti-his-tag Western blot (primary antibody: mouse anti-4H, Quiagen, secondary rabbit anti-mouse IgG-HRP, Sigma-Aldrich). OD_600_-normalized periplasmic extracts were prepared from replicate, overnight induced shake flask cultures (SF). Similar sample volumes were loaded in each lane. Inactive PglB_mut_: pGVXN115; wild-type PglB: pGVXN970; PglB N311V: pGVXN1217. Host strain: *S. enterica* SGSC228 (pMIK44). Expected size of AcrA-6H: 40 kDa.

wild-type PglB

PglB N311V

PglB mut

(*a*)

SF1 SF2

SF1 SF2

kDa

170

130

100

70

55

40

35


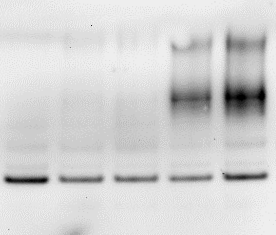


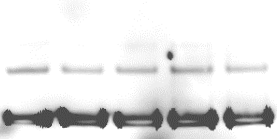


(*b*)

SF1 SF2

PglB N311V

wild-type PglB

SF1 SF2

PglB mut

kDa

170

130

100

70

55

40

35
