# Supplementary material for: Increased efficiency of Campylobacter jejuni N-oligosaccharyltransferase PglB by structure-guided engineering
Source: Open Biol. 2015 Apr 1;5(4):140227. doi: 10.1098/rsob.140227 (PMC4422122; doi:10.1098/rsob.140227)
Supplement: Experimental_details.docx [file rsob140227supp2.docx]

**Experimental details on plasmids, *pglB* mutant libraries and bacterial strains**

1. *Construction of plasmids*

Plasmids used in this study are listed in Supplementary Table ST1. Plasmid pGVNX1050 was constructed by PCR-subcloning of codon-optimized pglB-HA from pGVXN925 into pACT3Kan, using restriction sites *Kpn*I and *Bam*HI, followed by insertion of a TAA stop codon in front of the sequence encoding the HA peptide tag by QuikChange. A size-reduced, recombined derivative of pGVNX1050 which lacked the *lacI* repressor gene (pGVXN1415, semi-constitutive expression of PglB) was isolated from a saturation mutagenesis library of pGXVN1050 and harbored the mutations PglB N311V-A669V. Loss of *lac*I led to a two-fold increase in CP5-EPA levels in addition to the beneficial effect of N311V (data not shown). In order to avoid any further recombination events and profit from the enhancing effect of the absence of *lac*I, pGVXN1413 was used for construction of mutant libraries. The latter plasmid, encoding wild-type PglB, was constructed by ligating a *Kpn*I-*Bam*HI fragment of pGVXN1050 (full-length, wild-type PglB) with a *Bam*HI-*Kpn*I vector backbone fragment of pGVXN1415. Template plasmid pGVXN1418 (PglB N311V) for second-round and third round libraries was constructed by ligation of an *Asc*I-*Bam*HI fragment of pGVXN1050 (last third of wild-type *pglB* gene) with a *Bam*HI-*Asc*I fragment of pGVXN1415. Size-reduced, pACT3 derived plasmids for expression of HA-tagged PglB and PglB N311V (pGVXN1929 and pGVXN1930) were constructed by removal of the TAA stop codon in front of the HA tag sequence in pGVXN1413 and pGVXN1418, respectively, using the QuikChange procedure. All plasmids were validated by DNA sequencing.

1. *Construction of libraries by saturation mutagenesis*

Saturation mutagenesis libraries, randomizing individual amino acid residues of PglB, were constructed by QuikChange as decribed previously (1). In the following the sequences of forward primers used in QuikChange reaction are given. Corresponding reverse complement sequences were used for reverse primers. Degenerate codons at mutated positions are underlined. Saturation mutagenesis of N311: 5’-GC TTC ATG TAC TTC AAC GTT NNK CAG ACG ATC CAA GAA GTG G-3’, saturation mutagenesis of Y77: 5’-CAT CAG CCG AAC GAT CTG AGT NNK TAC GGT AGC TCT CTG TCC G-3’, saturation mutagenesis of S80: 5’-CG AAC GAT CTG AGT TAT TAC GGT NNK TCT CTG TCC GCG CTG ACC-3’, saturation mutagenesis of Q287: 5’-GGT GTT GAT CCG ATT CTG TAC NNK CTG AAA TTT TAT ATC TTC CGC TCA G-3’, saturation mutagenesis of L288: 5’-GTT GAT CCG ATT CTG TAC CAG NNK AAA TTT TAT ATC TTC CGC TCA GAT G-3’, saturation mutagenesis of K289: 5’-GAT CCG ATT CTG TAC CAG CTG NNK TTT TAT ATC TTC CGC TCA GAT GAA TCG-3’, saturation mutagenesis of F290: 5’-CCG ATT CTG TAC CAG CTG AAA NNK TAT ATC TTC CGC TCA GAT GAA TCG-3’, saturation mutagenesis of Y291: 5’-CG ATT CTG TAC CAG CTG AAA TTT NNK ATC TTC CGC TCA GAT GAA TCG-3’, saturation mutagenesis of R294: 5’-G TAC CAG CTG AAA TTT TAT ATC TTC NNK TCA GAT GAA TCG GCA AAC CTG-3’. First round libraries were constructed using pGVXN1413 as template. Second round libraries were constructed using pGVXN1418 (PglB N311V) or pGVXN1930 (PglB-HA N311V) as template. Commerically available, ultracompetent *E. coli* XL10-Gold were used for transformation of QuikChange reactions.

1. *Construction of a shuffled library*

A shuffled library of neutral and slightly beneficial amino acid substitutions derived from second round libraries (Table 1) was constructed with the Multi Site-Directed Mutagenesis kit according to the manufacturer’s instructions (Stratagene). A mix of three oligonucleotides was used, primer 5’- CAT CAG CCG AAC GAT CTG AGT YMT TAC GGT MGT TCT CTG TCC GCG CTG AC-3’ targeting the Y77 region and a mix (4:1 molar ratio) of primers 5’- C GGT GTT GAT CCG ATT CTG TAC MVG WTK MAK TTT TAT ATC TTC CGC TCA GAT GAA TCG -3’ and 5’- C GGT GTT GAT CCG ATT CTG TAC MVG WTK CGT TTT TAT ATC TTC CGC TCA GAT GAA TCG -3’ targeting the EL5 region. Improved PglB variant N311V (pGVXN1418) was used as template. The following amino acids were encoded by the mutagenic primers: Position PglB*_C.jejuni_* Tyr77 – wt Tyr, His, Pro, Ser (wobble codon YMT, Y = C/T, M = A/C); position Ser80 – wt Ser, Arg (wobble codon MGT, M = A/C); position Gln287 – wt Gln, Pro, Arg, Lys, Thr (wobble codon MVG, M = A/C, V = A/C/G); position Leu288 – wt Leu, Phe, Met, Ile (wobble codon WTK, W = A/T, K = G/T); position Lys289 – wt Lys, Asn, His, Gln, Arg (wobble codon MAK, M = A/C, K = G/T, codon CGT encoded by second primer).

**Suppl. Table ST1. Strains and plasmids used in this study.**

| **Strain / plasmid** | **Description** | **Marker** | **Reference** |
| --- | --- | --- | --- |
| *Bacterial strains* |  |  |  |
| *E. coli* XL10-Gold | Tet^R^ Δ(*mcrA*)*183* Δ(*mcrCB-hsdSMR-mrr*)*173 endA1 supE44 thi-1 recA1 gyrA96 relA1 lac* Hte [F´ *proAB lacI*q*Z*Δ*M15* Tn*10* (Tet^R^) Amy Cam^R^] | Cm^R^ | Stratagene |
| *E. coli* DH5α | K-12 φ80d*lacZΔM15* *endA1 recA1 hsdR17*(rK−mK+) *supE44 thi-1 gyrA96 relA1* *Δ(lacZYA-argF)U169 F−* | - | Clontech |
| *E. coli* CLM24 | W3110 Δ*waaL* | - | [1] |
| *E. coli* StGVXN1717 | W3110 Δ*waaL* Δ*wecA-wzzE* Δ*rmlB-wecG*::cat | Cm^R^ | [2] |
| *S. enterica* SGSC228 | sv. Typhimurium LT2; *waaL*446 | - | [3] |
| *Plasmids* |  |  |  |
| pACT3Kan | Medium copy number vector for IPTG-inducible expression; *lacI*, *P_tac_*, ori: pACYC184/p15a | Kan^R^ | [4] |
| pEXT21 | Low copy number vector for IPTG-inducible expression; *lacI*, *P_tac_*, ori: IncW | Sp^R^ | [5] |
| pACYC(*pgl*_mut_) | *C. jejuni* heptasaccharides, constitutive expression; *pgl* operon of *C. jejuni* with inactive PglB variant W458A-D459A (PglB_mut_), ori: pACYC184/p15a | Cm^R^ | [6] |
| pMIK44 | *C. jejuni* AcrA-6H with two native and one engineered glycosylation site (T127D), L-arabinose inducible expression. N-terminal ssPelB signal sequence for secretion to periplasm. High copy number plasmid. Ori: pBR322 | Amp^R^ | [7] |
| pGVXN115 | PglB_mut_, IPTG inducible expression; pEXT21 vector (ori: IncW) | Sp^R^ | [8] |
| pGVXN150 | *Pseudomonas aeruginosa* exotoxoid A (EPA) with 2 engineered N-glycosylation sites, L-arabinose inducible expression; N-terminal ssDsbA signal peptide for secretion to periplasm and C-terminal 6H tag, ori: pBR322 | Amp^R^ | [8] |
| pGVXN393 | *Staphylococcus aureus* CP5 capsular polysaccharides, constitutive expression; ori: IncPa | Tet^R^ | [2] |
| pGVXN408 | Inactive *C. jejuni* PglB-HA W458A-D459A (PglB_mut_), IPTG inducible expression; pACT3Kan vector | Kan^R^ | [4] |
| pGVXN925 | *C. jejuni* PglB with C-terminal hemagglutinin (HA) tag, codon-optimized for *E. coli*; high copy number cloning vector pUC57 | Amp^R^ | GenScript |
| pGVXN970 | wild-type, untagged PglB*_Cj_*, codon-optimized for *E. coli*, IPTG inducible expression; pEXT21 vector | Sp^R^ | GenScript |
| pGVXN1050 | wild-type, untagged PglB*_Cj_*, codon-optimized for *E. coli*, IPTG inducible expression; pACT3Kan vector | Kan^R^ | This study |
| pGVXN1217 | PglB*_Cj_* N311V, derivative of pGVXN970 constructed by site-directed mutagenesis | Sp^R^ | GenScript |
| pGVXN1413 | wild-type PglB*_Cj_*, semi-constitutive expression; size-reduced pACT3Kan vector without *lacI*, derivative of pGVXN1415 | Kan^R^ | This study |
| pGVXN1415 | PglB*_Cj_* N311V-A669V, semi-constitutive expression; size-reduced pACT3Kan vector without *lacI,* isolated from a saturation mutagenesis library of pGVXN1050 | Kan^R^ | This study |
| pGVXN1418 | PglB*_Cj_* N311V, derivative of pGVXN1415 | Kan^R^ | This study |
| pGVXN1929 | HA-tagged, wild-type PglB*_Cj_*, derivative of pGVXN1413 | Kan^R^ | This study |
| pGVXN1930 | HA-tagged, wild-type PglB*_Cj_*, derivative of pGVXN1418 | Kan^R^ | This study |
| pGVXN1942 | PglB*_Cj_* S80R-Q287P-N311V, isolated from a shuffled library, derivative of pGVXN1418 | Kan^R^ | This study |

**References**

1. Feldman MF, Wacker M, Hernandez M, Hitchen PG, Marolda CL, Kowarik M, Morris HR, Dell A, Valvano MA, Aebi M. 2005 Engineering N-linked protein glycosylation with diverse O antigen lipopolysaccharide structures in *Escherichia coli*. *Proc. Natl. Acad. Sci. USA*. **102**, 3016-3021. (doi:10.1073/pnas.0500044102)

2. Wacker M, Wang L, Kowarik M, Dowd M, Lipowsky G, Faridmoayer A, Shields K, Park S, Alaimo C, Kelley KA *et al.* 2014 Prevention of *Staphylococcus aureus* infections by glycoprotein vaccines synthesized in *Escherichia coli*. *J. Infect. Dis.* **209**, 1551-1561. (doi:10.1093/infdis/jit800)

3. Wacker M, Feldman MF, Callewaert N, Kowarik M, Clarke BR, Pohl NL, Hernandez M, Vines ED, Valvano MA, Whitfield C *et al.* 2006 Substrate specificity of bacterial oligosaccharyltransferase suggests a common transfer mechanism for the bacterial and eukaryotic systems. *Proc. Natl. Acad. Sci. USA*. **103**, 7088-7093. (doi:10.1073/pnas.0509207103)

4. Ihssen J, Kowarik M, Wiesli L, Reiss R, Wacker M, Thöny-Meyer L. 2012 Structural insights from random mutagenesis of *Campylobacter jejuni* oligosaccharyltransferase PglB. *BMC Biotechnol.* **12**, 67. (doi:10.1186/1472-6750-12-67)

5. Dykxhoorn DM, StPierre R, Linn T. 1996 A set of compatible tac promoter expression vectors. *Gene* **177**, 133-136.

6. Wacker M, Linton D, Hitchen PG, Nita-Lazar M, Haslam SM, North SJ, Panico M, Morris HR, Dell A, Wren BW *et al.* 2002 N-linked glycosylation in *Campylobacter jejuni* and its functional transfer into *E. coli*. *Science*. **298**, 1790-1793. (doi:10.1126/science.298.5599.1790)

7. Kowarik M, Young NM, Numao S, Schulz BL, Hug I, Callewaert N, Mills DC, Watson DC, Hernandez M, Kelly JF *et al.* 2006 Definition of the bacterial N-glycosylation site consensus sequence. *EMBO J.* **25**, 1957-1966.

8. Ihssen J, Kowarik M, Dilettoso S, Tanner C, Wacker M, Thöny-Meyer L. 2010 Production of glycoprotein vaccines in *Escherichia coli*. *Microb. Cell Fact*. **9**, 61. (doi:10.1186/1475-2859-9-61)
